# The Effects of Repeated Testing, Simulated Malingering, and Traumatic Brain Injury on Visual Choice Reaction Time

**DOI:** 10.3389/fnhum.2015.00595

**Published:** 2015-11-24

**Authors:** David L. Woods, John M. Wyma, E. W. Yund, Timothy J. Herron

**Affiliations:** ^1^Human Cognitive Neurophysiology Laboratory, Veterans Affairs Northern California Health Care System, MartinezCA, USA; ^2^UC Davis Department of Neurology, SacramentoCA, USA; ^3^Center for Neurosciences, University of California, DavisDavis, CA, USA; ^4^UC Davis Center for Mind and Brain, DavisCA, USA

**Keywords:** aging, concussion, head injury, reliability, response selection, feigning, effort, timing precision

## Abstract

Choice reaction time (CRT), the time required to discriminate and respond appropriately to different stimuli, is a basic measure of attention and processing speed. Here, we describe the reliability and clinical sensitivity of a new CRT test that presents lateralized visual stimuli and adaptively adjusts stimulus onset asynchronies using a staircase procedure. Experiment 1 investigated the test–retest reliability in three test sessions performed at weekly intervals. Performance in the first test session was accurately predicted from age and computer-use regression functions obtained in a previously studied normative cohort. Central processing time (CentPT), the difference between the CRTs and simple reaction time latencies measured in a separate experiment, accounted for 55% of CRT latency and more than 85% of CRT latency variance. Performance improved significantly across the three test sessions. High intraclass correlation coefficients were seen for CRTs (0.90), CentPTs (0.87), and an omnibus performance measure (0.81) that combined CRT and minimal SOA *z*-scores. Experiment 2 investigated performance in the same participants when instructed to feign symptoms of traumatic brain injury (TBI): 87% produced abnormal omnibus *z*-scores. Simulated malingerers showed greater elevations in simple reaction times than CRTs, and hence reduced CentPTs. Latency-consistency *z*-scores, based on the difference between the CRTs obtained and those predicted based on CentPT latencies, discriminated malingering participants from controls with high sensitivity and specificity. Experiment 3 investigated CRT test performance in military veterans who had suffered combat-related TBI and symptoms of post-traumatic stress disorder, and revealed small but significant deficits in performance in the TBI population. The results indicate that the new CRT test shows high test–retest reliability, can assist in detecting participants performing with suboptimal effort, and is sensitive to the effects of TBI on the speed and accuracy of visual processing.

## Introduction

Choice reaction times (CRTs) have been widely used to quantify attention and processing speed in clinical populations, including patients with head injury (Stuss et al., 1989b; Bashore and Ridderinkhof, 2002; Iverson et al., 2005), post-traumatic stress disorder (PTSD; Ponsford et al., 2012), multiple sclerosis (Snyder et al., 2001), Parkinson’s disease (Papapetropoulos et al., 2010), and schizophrenia (Pellizzer and Stephane, 2007). The clinical utility of a CRT test depends upon its reliability (Gualtieri and Johnson, 2006), precision (Plant and Quinlan, 2013), and sensitivity (Collins and Long, 1996). In Experiment 1, we describe the reliability and precision of a new CRT test (Woods et al., 2015c); in Experiment 2, we investigate its sensitivity in detecting participants feigning cognitive impairments; and in Experiment 3, we investigate its sensitivity in detecting processing-speed deficits among patients with combat-related traumatic brain injury (TBI) and PTSD.

## Experiment 1: Test–Retest Reliability and Learning Effects

**FIGURE 1:**
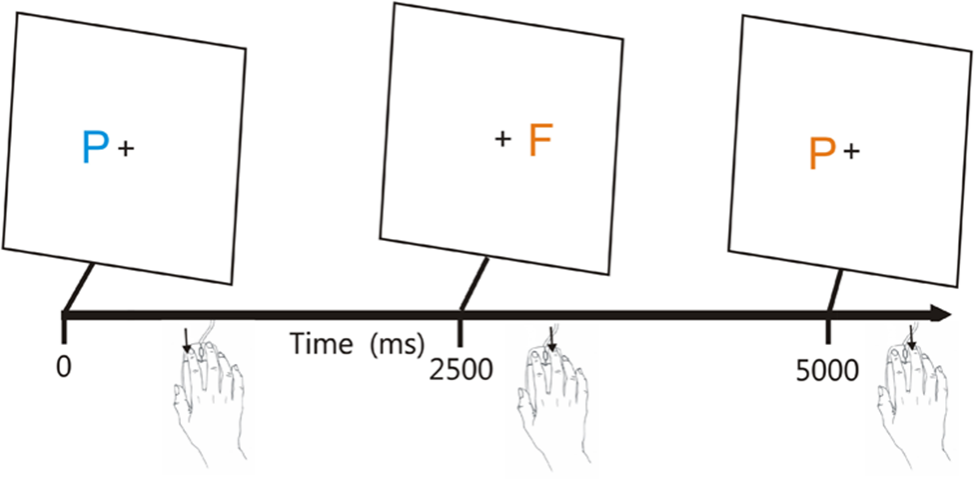
**The visual feature conjunction task.** Subjects performed a visual feature conjunction task with colored letters (blue P, blue F, orange P, or orange F) subtending 0.5° of visual angle randomly presented to the left or right hemifield, 1.6° from the fixation cross. Stimulus durations were 200 ms. Right-handed subjects pressed the left mouse button for targets (blue P’s, probability 40%) and pressed the right mouse button for non-targets, i.e., letters which resembled the target in color, shape, or neither feature (probability 20% each). The response button could be spatially compatible (trials 1 and 2) or spatially incompatible (trial 3) with the stimulus visual field. Stimulus onset asynchronies (SOAs) began at 2500 ms and were either reduced by 3% following each pair of successive hits or increased by 3% following each miss. From Woods et al. (2015c).

The CRT test incorporates a rapid serial visual feature-conjunction task in which participants press one mouse button in response to a target letter (blue P) and press the other mouse button to respond to non-target letters that share color (blue F), shape (orange P), or neither target feature (orange F; see **Figure 1**). Stimulus onset asynchronies (SOAs) are adjusted adaptively based on participant performance and stimuli are delivered randomly to the left or right hemifield to evaluate lateralized processing deficits.

Two previous large-scale studies showed that CRT latencies increase markedly with age (at 2.8 ms/year; Woods et al., 2015c) and vary systematically with the type of stimulus presented: CRT latencies are 40 ms faster to the more easily discriminated non-target stimuli with no target features than to non-target stimuli that share the color or shape of the target. In addition, latencies are reduced when stimuli and responses are spatially compatible, i.e., when the mouse button used for responding is ipsilateral to the hemifield of stimulus presentation (Klein and Ivanoff, 2011).

Experiment 1 first compared the age- and computer-use regressed *z*-scores in a group of 46 young participants with the results of a large normative control population tested previously (Woods et al., 2015c). The central question was whether the age and computer-use regression functions established in the normative population would apply accurately to the younger participants.

Experiment 1 next examined the test–retest reliability of the CRT measures by repeating two more test sessions at weekly intervals. Previous studies have found high to moderately high test–retest reliability for CRT latency measures. For example, Lemay et al. (2004) tested 14 older subjects three times at 14-day intervals and found intraclass correlation coefficients (ICCs) of 0.79. Straume-Naesheim et al. (2005) examined CRTs in 271 elite Norwegian soccer players using the CogSport battery and found an ICC of 0.65, with lower ICCs seen for trial-to-trial variance (0.39) and accuracy (0.14) measures. Collie et al. (2003b) reported ICCs of 0.69 for CogState CRT latency measures among 60 subjects tested at weekly intervals, with lower ICCs (0.51) again seen for accuracy. Lapshin et al. (2013) found ICCs of 0.85 for CRT latency measures in a mixed population of 30 patients with multiple sclerosis and 19 controls, while Lee et al. (2012) found ICCs of 0.58 in 254 children tested at a 2-month interval.

Performance improvements have also been reported in repeated test sessions in some studies (Collie et al., 2003a; Straume-Naesheim et al., 2005), but not in others (Lemay et al., 2004; Lee et al., 2012). In Experiment 1, we investigated whether performance improvements occurred across repeated tests, and, in addition, examined whether greater performance improvements were observed for more difficult to discriminate stimuli and for spatially incompatible stimulus-response pairings.

### Methods

#### Participants

The demographic characteristics of the participants in Experiment 1 are shown in **Table 1**, along with the characteristics of the previously studied normative sample, whose results have been presented in more detail elsewhere (Woods et al., 2015c). The 46 young volunteers (mean age = 26.2 years) were recruited from internet advertisements. The group as a whole was very well-educated (average of 15.0 years of education), with many of the younger participants still enrolled in college. Ethnically, 68% were Caucasian, 11% Latino, 9% African American, 10% Asian, and 2% other. The participants were required to meet the following inclusion criteria: (a) fluency in the English language; (b) no current or prior history of bipolar disorder, mania, or schizophrenia; (c) no current substance abuse; (d) no concurrent history of neurologic disease known to affect cognitive functioning; (e) auditory functioning sufficient to understanding normal conversational speech and visual acuity normal or corrected to 20*/*40 or better. The participants also completed a brief questionnaire that obtained information about their age, educational attainment, and daily hours of computer-use. All participants signed written consent forms approved by the institutional review board (IRB) at the Veterans Affairs Northern California Health Care System (VANCHCS) and were compensated for their participation. Participants underwent three test sessions at weekly intervals.

**Table 1:** Participants in the experiments.

Experiment	Group	*N*	Ages	Education	Male (%)
Woods et al., 2015c (Experiment 1)	Norms	1467	18-65; 46.3 (11.6)	6-20; 12.5 (3.2)	40.1
Experiments 1 and 2	Control/malinger	46	18-46; 26.2 (5.4)	12-18; 15.0 (2.0)	54.3
Experiment 3	mTBI	22	20-61; 33.4 (11.5)	10-18; 13.6 (5.8)	100.0
	sTBI	4	35-57; 46.0 (9.0)	12-16; 13.5 (1.9)	75.0

*Norms, control subjects from a previous normative study using the same paradigm; Malinger, Experiment 1 control participants instructed to malinger in Experiment 2. mTBI, mild TBI; sTBI, severe TBI. The range, mean, and variance are shown for age and education.*

#### Materials and Procedure

Choice reaction time testing required 4–6 min per participant and occurred after simple reaction time (SRT) testing, mid-way through a battery of cognitive tests.^1^ Testing was performed in a quiet room using a standard personal computer (PC) controlled by Presentation^®^ software (Versions 13 and 14, NeuroBehavioral Systems, Berkeley, CA, USA).

**Figure 1** shows the paradigm.^2^ Participants responded to the target (blue P, probability = 40%) by pressing the left mouse button, and responded to the three different non-target stimuli (probability 20% each) by pressing the right mouse button. The response buttons were reversed for left-handed participants. The letters P and F appeared in blue or orange colors (selected to reduce the influence of possible dichromatic anomalies), with non-target stimuli differing from the target in color (orange P), shape (blue F), or both features (orange P). Stimulus durations were fixed at 200 ms. Participants were trained to criterion levels (80% correct) in 20 practice trials, repeated if necessary, and then performed 140 test trials. SOAs were initially set at 2500 ms and were reduced by 3% following two successive correct responses and increased by 3% following each error or response omission.

#### Timing Precision

Hardware calibrations showed a delay of 11.0 ± 0.1 ms in the illumination of the 17” Samsung Syncmaster monitor when measured with a photodiode. Delays associated with the high-speed computer-gaming mouse (Razer Sidewinder, Carlsbad, CA, USA) were measured with an electronic relay and showed a mean of 6.8 ± 1.8 ms. Thus, mean hardware delays totaled 17.8 ms.

Presentation software reports timing-uncertainty measures for each event to quantify the timing precision as tests are performed. The occurrence of each event is associated with three different event times measured with the high-precision (100 kHz) programmable clock: T_0_, the last reported time before the event occurred; T_1_, the time of the event-monitoring loop associated with event occurrence; and T_2_, the time following the timing loop in which the event occurrence was first noted. While the event may have occurred before the time T_1_, Presentation may have read the time (T_1_) before testing for the response in the loop cycle. As a result, the time T_2_ must be used to define the upper limit of event latency. For example, consider the case where Presentation determined that no response was evident in a loop at 480.0 ms (T_0_), a loop at 480.1 ms (T_1_) was associated with a response, and a loop at 480.2 ms (T_2_) occurred after the response. These times establish a time range, T_2_-T_0_, which includes a time, T_0_ (480.0 ms), that was definitely before the response, and a time, T_2_ (480.2 ms), that was definitely after the response. Therefore, Presentation reports the response latency as T_0_ (480.0 ms) with an associated time uncertainty of T_2_-T_0_ (0.2 ms).

While Presentation software minimizes interruptions due to disk access and computational operations intrinsic to the test by interleaving these operations with the high-speed event-timing loop, timing errors can still occur if the event-timing loop is interrupted by resource-demanding operations (e.g., reading or writing from disk) or operating system processes. For example, if a 10 ms operating system interruption occurred at the instant that the subject responded in the trial described above, the CRT latency reported would be unchanged (480.0 ms), but the event-time uncertainty T_2_-T_0_ would be increased by 10 ms.

The event-time uncertainties reported by Presentation for 10,379 stimulus events in Experiment 1 showed a mean of 0.12 ms (±0.34), with three stimuli showing event-time uncertainties greater than 1.0 ms, including one instance where stimulus-time uncertainty was 18.2 ms. The time uncertainties reported for 7,353 response events showed a mean of 0.24 ms (±0.67), with 17 responses showing timing uncertainties greater than 1.0 ms, including one instance where response-time uncertainty was 41.5 ms. In summary, these results indicate that software timing was extremely precise, and software timing errors had minimal influence on the results.

#### Data Analysis

We quantified mean CRTs along with intrasubject (trial-to-trial) CRT standard deviations and hit rates for each type of stimulus. A response window of 250–1250 ms was used: failure to generate a response during this interval was categorized as a miss, and an incorrect response occurring within the window was categorized as an error. The minimum SOA (mSOA), which reflected the ability of participants to accurately respond to stimuli presented at rapid rates, was also measured for each participant. In cases where SOAs were reduced below 1250 ms, multiple responses could occur within a response window. In these cases, responses were assigned to stimuli in the order in which they occurred.

#### Statistical Analysis

Overall performance was examined using mean CRTs and central processing time (CentPT), the difference between CRTs and SRTs measured in a companion test performed on the same day (Woods et al., 2015a). In addition, we analyzed intrasubject (trial-to-trial) standard deviations and the coefficient of variation (CV, i.e., trial-by-trial standard deviations divided by the mean CRT). Since the distribution of minimal SOAs was significantly skewed, minimal SOAs were log-transformed (log-mSOA) before further analysis.

Previous large-scale studies showed a large effect of age on performance, but minimal effects of education (Woods et al., 2015c). The reanalysis of these results revealed that CRT latencies were also reduced in participants with greater daily computer-use [*r* = -0.15, *t*(1464) = 4.80, *p* < 0.0001]. Multiple regression analysis showed that the combined influence of age and computer-use accounted for 22.5% of CRT variance, with both factors exerting significant effects [age *t*(1463) = 19.5, *p* < 0.0001; computer-use, *t*(1463) = -5.40, *p* < 0.0001]. Age and computer-use also had significant effects on CentPT latencies [age, *t*(1463) = 17.4, *p* < 0.0001; computer-use, *t*(1463) = -4.35, *p* < 0.0001]. Therefore, CRT and CentPT *z*-scores in the current study were calculated after regressing out the effects of age and computer-use on the observed CRT and CentPT latencies. In contrast, age and computer-use had little influence on log-mSOA measures, where they accounted for less than 1% of the variance. Therefore, no regressions were used when calculating log-mSOA *z*-scores. Finally, omnibus *z*-scores were used to summarize overall performance by adding CRT *z*-scores and log-mSOA *z*-scores, and then normalizing the resulting distribution.

The results were analyzed with analysis of variance (ANOVA) using CLEAVE (www.ebire.org/hcnlab). Greenhouse–Geisser corrections of degrees of freedom were uniformly used in computing *p*-values in order to correct for covariation among factors and interactions. Effect sizes are reported as Cohen’s *d* values or as $\omega_{p}^{2}$. Because of the large number of statistical comparisons, significance levels were set at 0.005. Correlation analysis was also used with significance levels evaluated with Student’s *t*-test. ICCs were calculated with SPSS (IBM, version 22).

### Results

**FIGURE 2:**
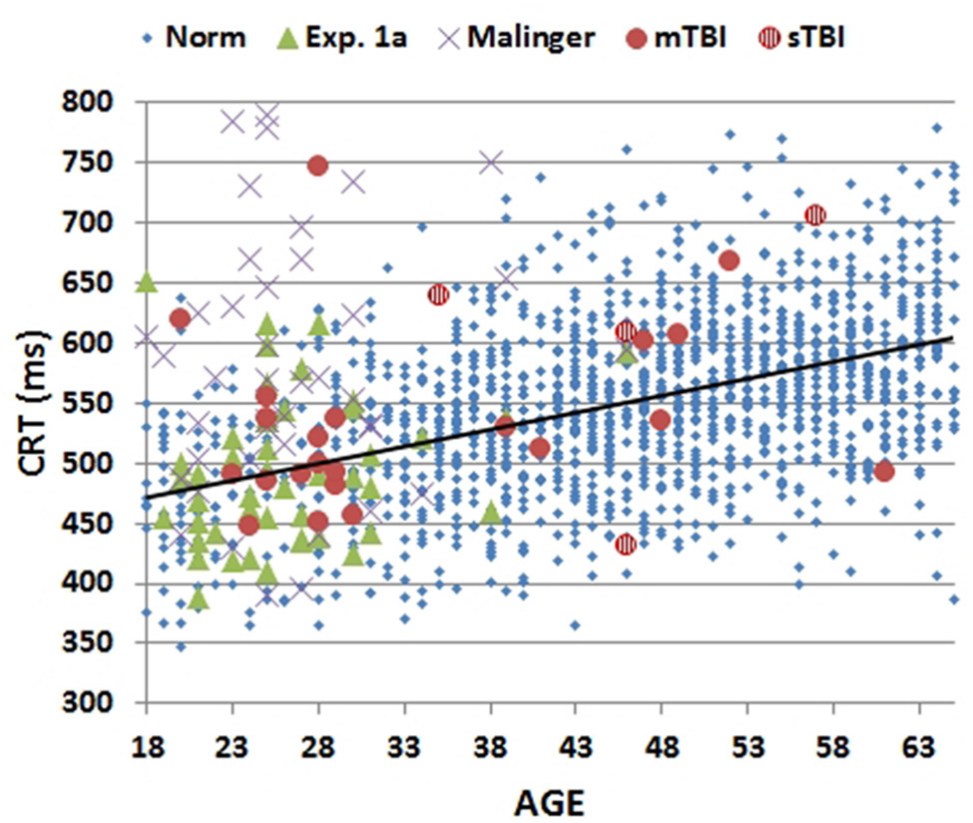
**Mean choice reaction times (CRTs) as a function of age.** CRTs were averaged over stimulus types for subjects of different ages from normative data (Norm), Experiment 1A, Experiment 2 (malinger), and Experiment 3 (TBI). The age-regression slope for the normative data is shown. CRTs for patients with mild TBI (mTBI, filled red circles) and severe TBI (sTBI, circles with vertical red stripes) are shown separately.

**FIGURE 3:**
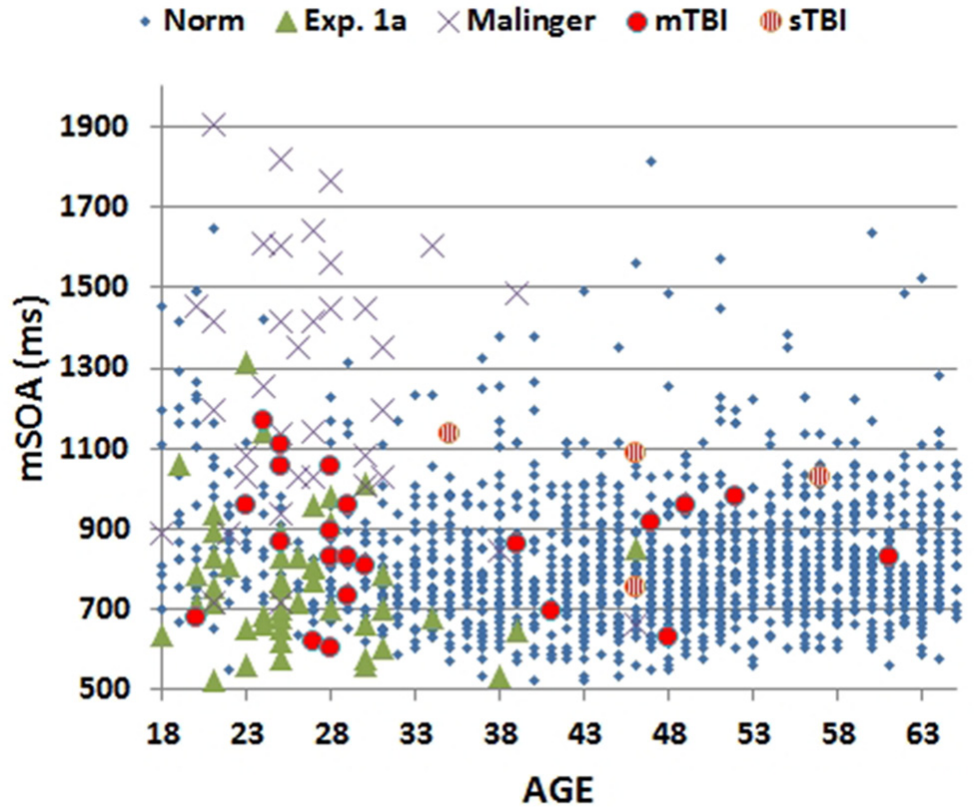
**Minimal stimulus onset asynchronies (mSOA) for subjects as a function of age.** Showing data from the normative study (Norm), Experiment 1A, Experiment 2 (Malinger), and Experiment 3 (TBI). mSOAs for patients with mild TBI (mTBI, filled red circles) and severe TBI (sTBI, circles with vertical red stripes) are shown separately.

**FIGURE 4:**
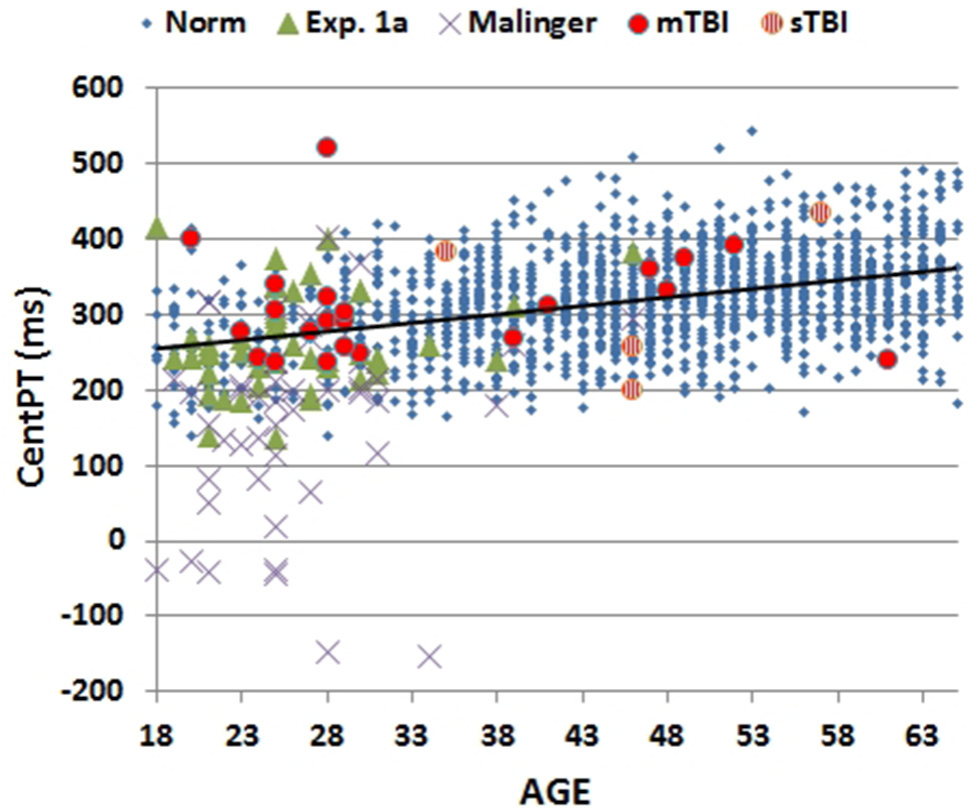
**Mean central processing times (CentPT) as a function of age.** CentPTs were averaged over stimulus types from control subjects in the normative study (Norm) and Experiment 1A, simulated malingerers (Experiment 2), and TBI patients (Experiment 3). The age-regression slope for the normative data is shown. The results from patients with mild TBI (mTBI, filled red circles) and severe TBI (sTBI, circles with vertical red stripes) are shown separately.

**FIGURE 5:**
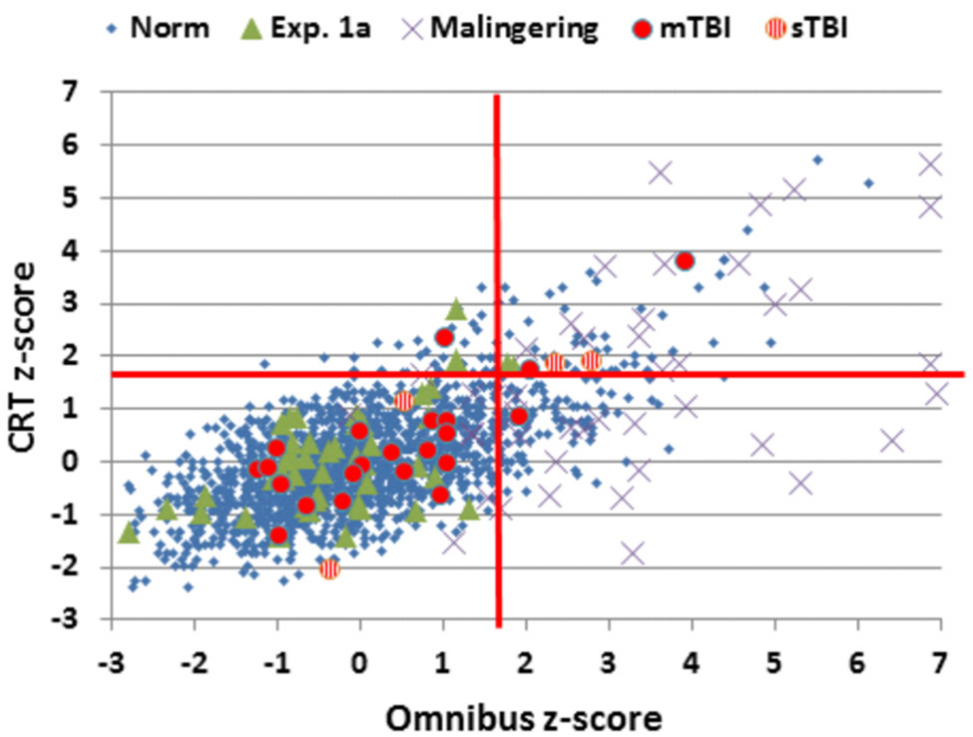
**Choice reaction time and Omnibus *z*-scores.** Data from control subjects in the normative data (Norm) and Experiment 1A, simulated malingerers (Experiment 2), and TBI patients (Experiment 3). *Z*-scores were calculated based on age- and computer-use regression slopes from the normative data. Patients with mild TBI (mTBI, filled red circles) and severe TBI (sTBI, circles with vertical red stripes) are shown separately. The data from four simulated malingerers with *z*-scores outside the range of the figure are not shown.

Mean CRT latencies from the individual participants in the first test session of Experiment 1 (1A) are shown as a function of age in **Figure 2** (green triangles), along with the results from the normative control population (blue diamonds). **Figure 3** shows a similar plot of mSOAs. **Figure 4** shows CentPTs as a function of age, and **Figure 5** shows the relationship between CRT and omnibus *z*-scores in the individual participants, after regressing out the effects of age and computer-use. CRTs in Experiment 1A averaged 494 ms, and mSOAs averaged 766 ms. A summary of the different measures is provided in **Table 2**.

**Table 2:** Results from Experiments 1 (three sessions), 2 and 3, and normative results from a previous experiment (Woods et al., 2015c).

Experiment	Norm	Experiment 1A	Experiment 1B	Experiment 1C	Experiment 2 Mal	Experiment 3 mTBI	Experiment 3 sTBI
Age	46.3	26.3	26.3	26.3	26.3	33.4	46.0
CRT	550	494	475	463	609	534	596
CRT (SD)	72.2	61.9	55.4	62.4	135.3	78.9	93.8
ISSD	162	148	154	150	195	169	172
CV (%)	29.5	29.7	32.2	32.1	32.2	31.8	28.9
mSOA	825	766	750	718	1579	866	1002
CentPT	319	263	244	234	158	311	318
CRT z	0.00	-0.01	-0.31	-0.50	1.85	0.32	0.69
L-mSOA z	0.00	-0.42	-0.56	-0.73	3.12	0.27	1.09
Omni z	0.00	-0.28	-0.60	-0.88	3.73	0.44	1.33
CentPT z	0.00	-0.20	-0.53	-0.70	-1.98	0.35	-0.04
LC z	0.00	0.12	0.18	0.11	8.64	-0.25	1.77

*Mal, malingering; CRT, mean choice reaction time; CentPT, central processing time (CRT-simple reaction time); ISSD, intrasubject standard deviation; CV, coefficient of variation; mSOA, minimum stimulus onset asynchrony. The bottom four rows show age- and computer-use regressed *z*-scores using parameters derived from the normative sample. z, *z*-score; L-mSOA, log-transformed minimum stimulus onset asynchrony; Omni, omnibus, the normalized sum of mSOA and CRT; LC, latency consistency. See **Table 1** for additional abbreviations.*

#### Comparisons with Normative Results

Age- and computer-use regressed CRT *z*-scores [-0.01] in Experiment 1A were virtually identical to those predicted by the age-regression functions from Woods et al. (2015c) [*F*(1,1510) = 0.01, NS]. Neither CentPT *z*-scores [-0.19, *F*(1,1510) = 1.77, NS] nor omnibus *z*-scores [-0.28, *F*(1,1510) = 3.45, *p* < 0.07] differed significantly from those in the normative group. However, there was a trend toward lower log-mSOA *z*-scores [-0.42, *F*(1,1510) = 7.71, *p* < 0.01].

#### Test–retest Reliability

**FIGURE 6:**
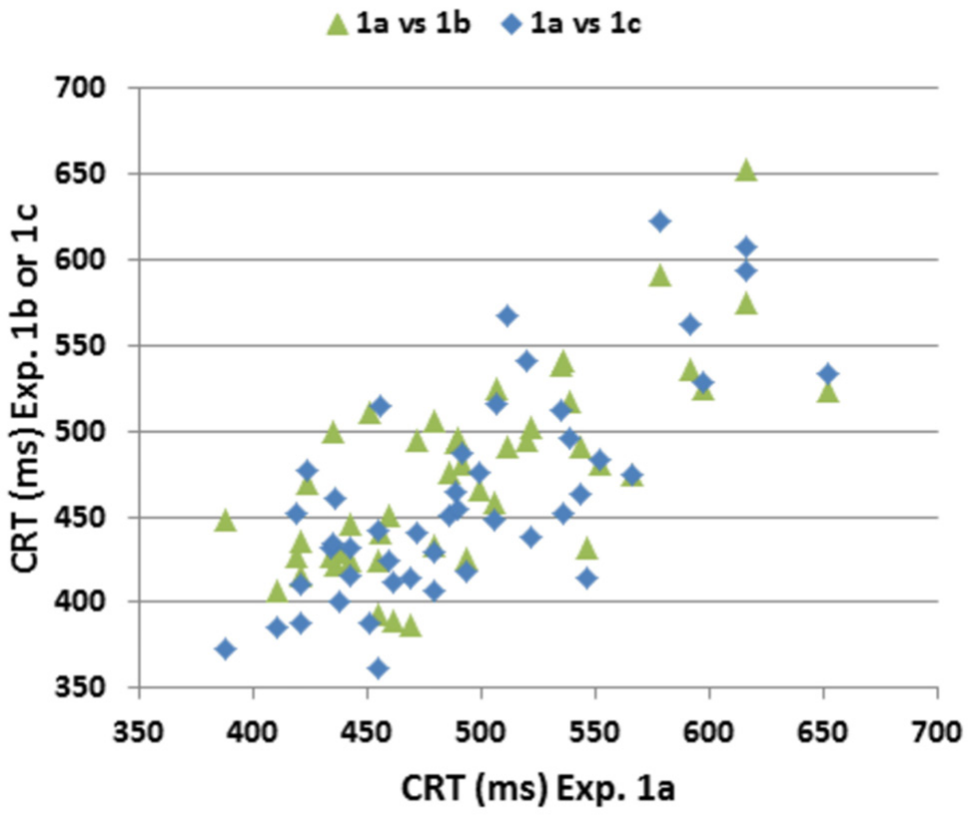
**Test–retest reliability of CRTs.** CRTs in Experiment 1a plotted against CRTs in Experiments 1B,C. Pearson correlations across repeated tests were 0.72 (Experiment 1A vs. Experiment 1B), 0.74 (Experiment 1A vs. Experiment 1C) and 0.77 (Experiment 1B vs. Experiment 1C).

**Figure 6** shows comparisons of CRT latencies across the three test sessions. ICCs across the three test sessions were used to evaluate test–retest reliability. ICCs were high for CRT *z*-scores (0.89), CentPT *z*-scores (0.86), and omnibus *z*-scores (0.81), while they were somewhat lower for log-mSOA *z*-scores (0.72), and considerably reduced for measures of trial-by-trial CRT variance (0.41) and the CV (0.27).

#### Stimulus and Spatial Compatibility Effects

An ANOVA for repeated measures was performed with the factors of Stimulus-Type (four different types), Stimulus-Response Spatial Compatibility (compatible or incompatible, see **Figure 1**), and Test Session (1, 2, or 3). There was a significant effect of Stimulus-Type on CRT latencies [*F*(3,132) = 40.48, *p* < 0.0001, $\omega_{p}^{2}$ = 0.50]: participants were faster to respond to distractors with no target features (455 ms) than to targets (474 ms), distractors with target color (500 ms), or distractors with target shape (486 ms). Hit rate showed corresponding variations with Stimulus-Type [*F*(3,132) = 20.63, *p* < 0.0001, $\omega_{p}^{2}$ = 0.33]: participants were more accurate when identifying distractors with no target features (96.6% correct) than when identifying targets (92.1%), distractors with target color (93.3%), or distractors with target shape (94.0%). Stimulus-Response Spatial Compatibility effects were also significant: CRTs were 28.5 ms faster [*F*(1,44) = 85.98, *p* < 0.0001, $\omega_{p}^{2}$ = 0.66] and 3.2% more accurate [*F*(1,44) = 34.96, *p* < 0.0001, $\omega_{p}^{2}$ = 0.44] when stimuli and responses were spatially compatible.

#### Learning Effects

Repeated testing (Experiments 1B,C) resulted in improvements in performance for CRT *z*-scores (-0.49), CentPT *z*-scores (-0.50), log-mSOA *z*-scores (-0.29), and omnibus *z*-scores (-0.60). Omnibus *z*-scores fell significantly with repeated testing [*F*(2,88) = 12.51, *p* < 0.0001, $\omega_{p}^{2}$ = 0.20], CRT latencies decreased [by 33 ms, *F*(2,88) = 14.60, *p* < 0.0001, $\omega_{p}^{2}$ = 0.24], and there was a trend toward reduced log-mSOAs [*F*(2,88) = 4.89, *p* < 0.02, $\omega_{p}^{2}$ = 0.08].

There was also a significant interaction between Test Session and Stimulus-Type [*F*(6,264) = 4.85, *p* < 0.0003, $\omega_{p}^{2}$ = 0.08]. This interaction reflected greater CRT latency reductions for the more difficult distractors (color -43 ms, shape -43 ms) in comparison to the distractors with no target features (-23 ms) and targets (-21 ms). In contrast, the magnitude of the spatial compatibility effect on CRT latencies was not influenced by repeated testing [*F*(2,88) = 0.98, NS].

Unlike CRT latencies, hit rate did not improve significantly over Sessions [*F*(2,88) = 1.78, NS], presumably because SOAs were reduced as performance improved, nor did Sessions interact significantly with the effect of Stimulus-Type on hit rate [*F*(6,264) = 0.53, NS]. However, there was a significant reduction in the magnitude of the Spatial Compatibility effect on hit rate over repeated sessions [from 4.2 to 1.0%, *F*(6,264) = 8.31, *p* < 0.0005, $\omega_{p}^{2}$ = 0.14].

### Discussion

#### Comparisons with Normative Data

The Experiment 1 data were well-fit by the regression functions derived from the normative population: CRT *z*-scores, log-mSOA *z*-scores, CentPT *z*-scores, and omnibus *z*-scores did not differ significantly between the participants in the first test session of Experiment 1 and the normative control group. This suggests that the regression functions relating age and computer-use in the normative population accurately corrected for the age and computer-use differences in the two populations.

#### Test–retest Reliability

In his discussion on the interpretation of results of neuropsychological tests, Iverson (2001) argued that clinically useful neuropsychological tests should show ICCs that exceed 0.75, citing the WAIS Processing Speed Index, where ICCs in excess of 0.75 were found in four of the six groups that underwent repeated testing during WAIS III normative data collection (Barr, 2003). For comparison, the manually administered NIH Processing Speed Toolbox showed an ICC of 0.72 in 89 adult controls (Weintraub et al., 2013; Carlozzi et al., 2014).

The ICCs obtained in Experiment 1 equaled or exceeded those reported for both manually administered tests of processing speed and previously reported computerized CRT tests (Versavel et al., 1997; Lemay et al., 2004; Straume-Naesheim et al., 2005; Falleti et al., 2006; Gualtieri and Johnson, 2006; Segalowitz et al., 2007; Resch et al., 2013). As in previous reports (Lemay et al., 2004; Straume-Naesheim et al., 2005), hit rate and trial-to-trial CRT variance measures showed lower ICCs than the CRT latencies.

#### Learning Effects and Test Complexity

Consistent with previous reports (Straume-Naesheim et al., 2005; Falleti et al., 2006; Rogers et al., 2013), we found significant reductions in CRT latencies and log-mSOAs over test sessions. The results contrast with the lack of learning effects in SRT paradigms (Woods et al., 2015a), and likely reflect the increased complexity of the CRT task. Further analysis revealed that repeated testing resulted in larger improvements in the speed and accuracy of processing harder-to-identify stimuli. The results also suggest that learning may influence the strength of stimulus-response spatial compatibility (i.e., the Simon effect) on hit rate by enabling more accurate responding to spatially incompatible stimulus-response pairs on repeated testing.

## Experiment 2: Simulated Malingering

Identifying participants who perform with suboptimal effort is a significant and growing challenge in neuropsychological testing (Clark et al., 2014). In particular, many patients with litigation or pension claims following head trauma show evidence of malingering on symptom-validity tests (Armistead-Jehle, 2010; McNally and Frueh, 2012). Previous studies have shown that subjects who are instructed to malinger on CRT tests show increased CRT latencies and reduced hit rates (Wogar et al., 1998; Bender and Rogers, 2004; Willison and Tombaugh, 2006). In Experiment 2, we retested the participants of Experiment 1 after giving them instructions to malinger, with the hypothesis that they would show a similar pattern.

In addition, we were also interested in investigating the extent to which malingering participants adjusted their performance so that the magnitude of abnormality on the CRT test resembled the magnitude of abnormality observed on a previously performed SRT test (Woods et al., 2015a). We reasoned that CRT vs. SRT comparisons might increase sensitivity to malingering because previous studies have found that malingering patients produce greater relative latency increases on SRT than CRT tests (Reicker, 2008), and sometimes even produce longer absolute SRT than CRT latencies (Kertzman et al., 2006). In contrast, patients with neurological disorders such as TBI show greater absolute and relative latency increases on CRT than SRT tests (Van Zomeren and Deelman, 1976; Willison and Tombaugh, 2006).

### Methods

#### Participants

The participants were identical to those of Experiment 1.

#### Materials and Procedures

The methods and procedures were identical to those of Experiment 1, but participants were given different instructions. After the third session of Experiment 1, participants were instructed to feign the symptoms of a patient with mild TBI during a fourth test session in the following week. The instructions, as described previously in Woods et al. (2015a,b), were as follows: “Listed below you’ll find some of the symptoms common after minor head injuries. Please study the list below and develop a plan to fake some of the impairments typical of head injury when you take the test. Do your best to make your deficit look realistic. If you make too many obvious mistakes, we’ll know you’re faking! Symptom list: difficulty concentrating for long periods of time, easily distracted by unimportant things, headaches and fatigue (feeling “mentally exhausted”), trouble coming up with the right word, poor memory, difficulty performing complicated tasks, easily tired, repeating things several times without realizing it, slow reaction times, trouble focusing on two things at once.”

#### Timing Precision

The hardware was identical to that used in Experiment 1. Event-time uncertainties for 9,479 stimulus presentations averaged 0.12 ms (±0.04 ms), with a maximum uncertainty of 0.8 ms. Event-time uncertainties for 6,903 responses averaged 0.18 ms (±0.12 ms), with seven events showing uncertainties greater than 1.0 ms, and a maximal uncertainty of 4.5 ms.

#### Latency-consistency *z*-scores

**FIGURE 7:**
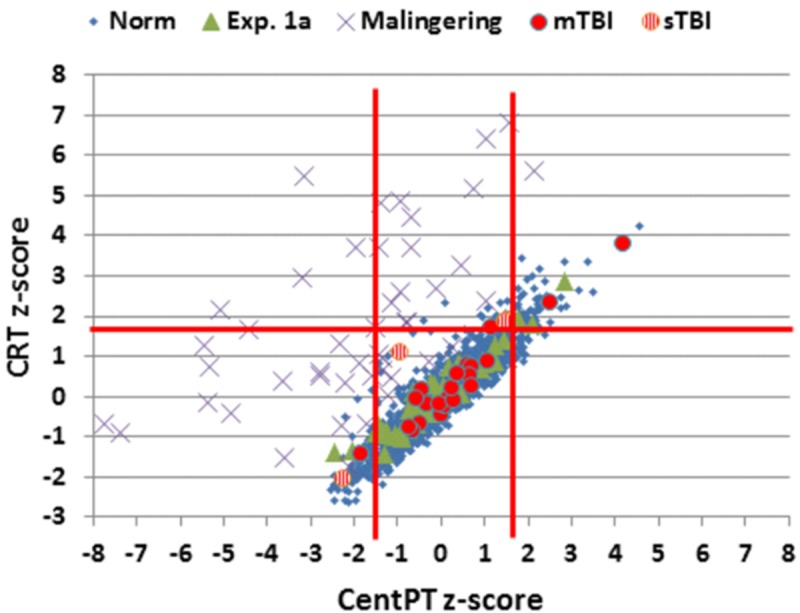
***Z*-scores for CentPT (central processing time) and CRT.** Data from controls in the normative study (Norm) and Experiment 1A, simulated malingerers, and patients with mild TBI (mTBI, filled red circles) and severe TBI (sTBI, circles with vertical red stripes). *Z*-scores were calculated using the age- and computer-use regression slopes from the normative data. The solid vertical red lines show limits for abnormally (*p* < 0.05) short and long CPTs.

In order to evaluate the consistency of performance on the CRT and SRT tests, we developed a latency-consistency *z*-score metric based on the relationship between CentPT *z*-scores and CRT *z*-scores in control participants. As seen in **Figure 7**, CentPTs were strongly correlated with CRTs in the normative population (*r* = 0.93) and the control participants in Experiment 1A (*r* = 0.96), so that CentPT latencies accounted for 86–92% of CRT latency variance. The difference between CentPT-predicted and observed CRTs was used to predict CRTs from CentPTs (CRT = 1.06^∗^CentPT + 213 ms), and the difference between observed and predicted CRTs was used to create *z*-scores. Latency-consistency *z*-scores in Experiment 1A averaged 0.12 (±0.73) and were not significantly different from those in the normative population (*z*-score = 0.0, by definition). We anticipated that simulated malingerers would show greater relative elevations in SRTs than CRTs, resulting in a reduction in CentPTs. As a result, the predicted CRT latencies based on CentPT latencies in malingerers would be reduced in comparison to the CRTs actually observed, resulting in elevated latency-consistency *z*-scores.

#### Statistical Analysis

The results were analyzed using ANOVA between groups when comparing the results with those of the normative controls. Other procedures were identical to those of Experiment 1.

### Results

The results from individual malingering participants (purple X’s) are included in **Figures 2–5** and summarized in **Table 2**. Omnibus *z*-scores in the malingering condition were significantly reduced in comparison to Experiment 1A [*F*(1,45) = 106.31, *p* < 0.0001, $\omega_{p}^{2}$ = 0.70], while CRT *z*-scores [*F*(1,45) = 26.11, *p* < 0.0001, $\omega_{p}^{2}$ = 0.36] and log-mSOA *z*-scores increased [*F*(1,45) = 106.31, *p* < 0.0001, $\omega_{p}^{2}$ = 0.70]. As predicted, CentPT *z*-scores were significantly reduced [*F*(1,45) = 29.14, *p* < 0.0001, $\omega_{p}^{2}$ = 0.38]. Elevations were also seen relative to the normative group for omnibus *z*-scores [*F*(1,1510) = 534.16, *p* < 0.0001, $\omega_{p}^{2}$ = 0.26], CRT *z*-scores [*F*(1,1510) = 127.93, *p* < 0.0001, $\omega_{p}^{2}$ = 0.08], and log-mSOA *z*-scores [*F*(1,1510) = 386.05, *p* < 0.0001, $\omega_{p}^{2}$ = 0.20], while CentPT *z*-scores were significantly reduced [*F*(1,1510) = 161.92, *p* < 0.0001, $\omega_{p}^{2}$ = 0.10]. Overall, 83% of malingering participants produced omnibus *z*-scores that were outside the normal (*p* < 0.05) range (**Figure 5**), including 46% with abnormal CRT *z*-scores and 70% with abnormal log-mSOA *z*-scores.

**FIGURE 8:**
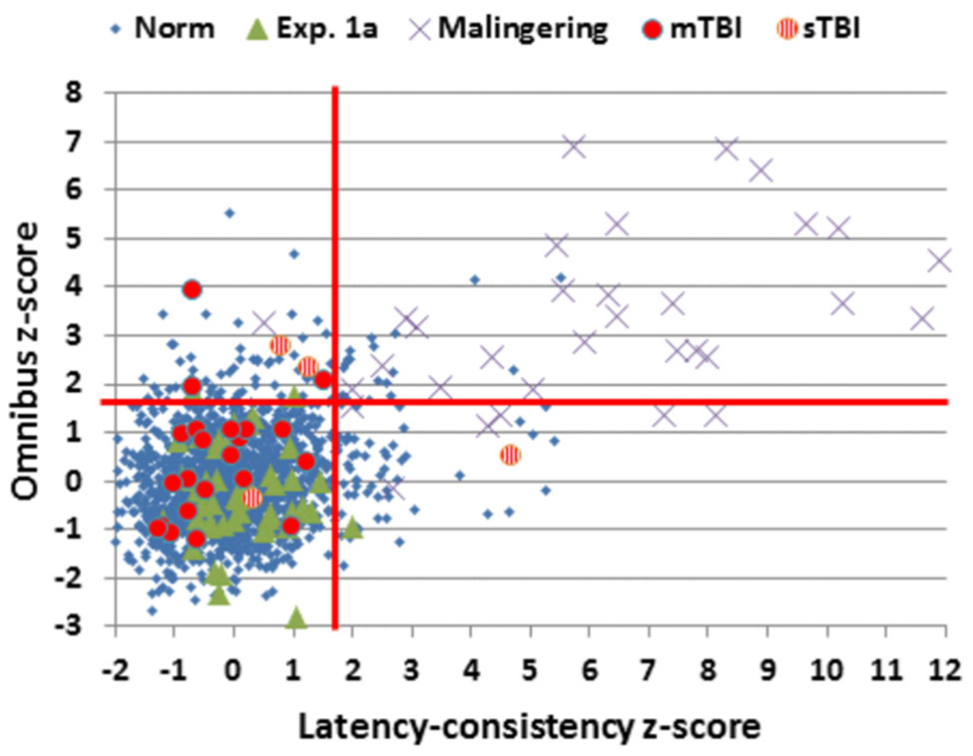
**Latency-consistency and Omnibus *z*-scores.** Data are shown from control subjects in the normative population (Norm) and Experiment 1A, simulated malingerers, and patients with mild TBI (mTBI, filled red circles) and severe TBI (sTBI, circles with vertical red stripes). The red lines show abnormality cutoffs (*p* < 0.05) based on the normative population. The data from 13 malingering subjects with latency-consistency *z*-scores > 12.0 are not shown.

Simulated malingerers showed greater slowing in the SRT than the CRT task, resulting in reductions in CentPTs (see **Figures 4** and **7**) and reduced correlations between CentPTs and CRTs [*r* = 0.57 vs. *r* = 0.93, *z* = 5.99, *p* < 0.0001]. Because CentPTs were reduced in simulated malingerers, predicted CRT latencies based on CentPT latencies (381 ms) were much shorter than those actually observed (609 ms). As a result, the mean latency-consistency *z*-score in simulated malingerers was 8.64, and, as shown in **Figure 8**, all but one of the simulated malingerers produced latency-consistency *z*-scores outside the normal (*p* < 0.05) range. With a more conservative latency-consistency *z*-score cutoff of 3.0, 87% of simulated malingerers showed abnormalities. In contrast, less than 1% of normal controls produced latency-consistency *z*-scores > 3.0, including fewer than 4% of control participants with abnormal Omnibus *z*-scores.

### Discussion

When instructed to feign symptoms of mild head injury, 83% of simulated malingerers produced abnormal omnibus *z*-scores, characterized by increased CRTs and reductions in hit rate associated with increased log-mSOAs. However, the relative increase in CRT latency was much less than the relative increase in SRT latency (Woods et al., 2015a), resulting in the paradoxical apparent increase in central processing speed (i.e., a reduction in CentPT) and large increases in latency-consistency *z*-scores.

A latency-consistency *z*-score cutoff of 3.0 had a sensitivity of 87% in detecting simulated malingerers, and a specificity greater than 99% for the entire control population (96% when only control participants with abnormal performance were considered). These abnormalities in latency-consistency *z*-scores reflected the inability of simulated malingerers to accurately adjust their performance “deficits” in SRT and CRT tests.

#### Simulated Malingering and Task Complexity

A number of previous studies have suggested that malingering effects are often reduced as task complexity increases. For example, Kertzman et al. (2006) examined SRTs and CRTs in schizophrenic prisoners suspected of malingering. In contrast to control subjects, the malingerers showed longer latencies on the SRT than CRT test. Wogar et al. (1998) examined the effects of malingering as a function of CRT test complexity. They used a match-to-sample task in which subjects were presented with letter strings above fixation and told to choose which of the two letter strings presented below fixation matched the target string. Letter strings ranged in length from one to ten letters. In one-letter displays, CRT latencies in a control group averaged 750 ms and increased linearly with the number of letters in the display with a slope of approximately 230 ms/letter. In contrast, in simulated malingerers, CRT latencies increased to 2,000 ms in one-letter conditions. However, the CRTs in simulated malingerers increased at the same rate as in control subjects with increasing list length. Thus, simulated malingerers showed substantial slowing in the easiest condition, but failed to adjust performance proportionally with increases in task difficulty. The results contrasted with those obtained from TBI patients, who showed increased CRT latencies in the one-letter condition and greater latency increases with increasing task complexity than either control or malingering participants.

Task-complexity effects in malingering participants may reflect the dual-task nature of malingering: malingerers must perform a primary task (i.e., the test) while simultaneously monitoring and adjusting performance to simulate impairment. As test difficulty increases, fewer cognitive resources are available to monitor and adjust performance. As a result, the relative magnitude of the malingering deficit decreases. For example, malingering effects are larger in the Trail Making Test, Part A than on the Trail Making Test, Part B (Haines and Norris, 2001; Egeland and Langfjaeran, 2007; Busse and Whiteside, 2012; Woods et al., 2015b). We found a similar effect here: malingering deficits were relatively larger in an SRT than CRT test.

#### Limitations

The participants in Experiment 2 had undergone three prior CRT test sessions before participating in the malingering experiment. Since repeated testing resulted in improvements in CRT performance, malingering effects would be somewhat larger when compared to “baseline” performance in session 3 than in session 1.

## Experiment 3. The Effects of Traumatic Brain Injury

Traumatic brain injury can result in impairments in attention and processing speed (Karr et al., 2014). For example, CRT latencies are increased following both mild TBI (mTBI) and severe TBI (sTBI) in the acute phase (Warden et al., 2001; Fong et al., 2009; Bryan and Hernandez, 2012), and remain delayed in the chronic phase following sTBI (Stuss et al., 1989a; Ferraro, 1996; Bashore and Ridderinkhof, 2002; Tombaugh et al., 2007). While processing speed usually recovers to normal levels in mTBI patients, the magnitude of processing speed deficits seen in the chronic phase of more severe TBI is one of the best predictors of ultimate functional outcome (Rassovsky et al., 2006). However, some mTBI patients show persistent CRT latency increases years after injury, although these are generally less prominent than the deficits of sTBI patients (Van Zomeren and Deelman, 1976; Stuss et al., 1989b; Tombaugh et al., 2007). Trial-to-trial CRT variance may also increase in both sTBI and mTBI patients (Stuss et al., 1989a; Collins and Long, 1996; Hetherington et al., 1996; Tombaugh et al., 2007). In Experiment 3, we evaluated the sensitivity of the CRT test in a mixed population of chronic mTBI and sTBI patients, with the hypothesis that we would observe more severe deficits following sTBI than mTBI.

### Methods

#### Participants

Twenty-eight Veterans with a history of TBI were recruited from the local VANCHCS patient population. The patients included 27 males and one female between the ages of 20 and 61 years (mean age = 35.8 years), with an average 13.6 years of education (**Table 1**) who had previously been evaluated with other computerized tests (Woods et al., 2011, 2015a,b,d; Hubel et al., 2013). All patients had suffered head injuries and transient loss or alteration of consciousness, and all were tested at least 1 year post-injury. Twenty four of the patients had suffered one or more combat-related incidents with a cumulative loss of consciousness of less than 30 min, no hospitalization, and no evidence of brain lesions on clinical MRI scans. These patients were categorized as mTBI. The remaining four patients had suffered severe accidents with hospitalization, brain abnormalities visible on neuroimaging, coma duration exceeding 8 h, and post-traumatic amnesia exceeding 72 h. These patients were categorized as sTBI. Evidence of PTSD, as reflected in elevated scores on the PTSD Checklist (PCL), was evident in 54% of the TBI sample. All patients signed written consent forms approved by the IRB at VANCHCS and were compensated for participation. They were informed that the studies were for research purposes only and that the results would not be included in their official medical records.

Two mTBI patients showed evidence of suboptimal effort, with latency-consistency *z*-scores of 13.8 and 12.7, respectively. These same two patients had shown evidence of suboptimal effort in other experiments performed during the same test session (Hubel et al., 2013; Woods et al., 2015a,b). Their data were therefore excluded from further analysis. Additional information about the severity and etiology of the TBIs in the remaining patients is included in **Table 3**.

**Table 3:** Traumatic brain injury (TBI) patient characteristics.

ID	Age	Edu	Etiology	TBI	PCL	mSOA (ms)	CRT (ms)
PAT001^c^	35	12	MVA	Severe	59	1138	639
PAT002^c,d^	24	12	Blast	Mild	54	1166	447
PAT003^c,d^	28	12	Blast	Mild	66	1058	746
PAT005^d^	46	12	MVA	Severe	42	1086	431
PAT012^c,d^	57	16	MVA	Severe	56	1031	705
PAT014	30	14	MVA	Mild	–	808	456
PAT038^c^	52	18	MVA	Mild	27	982	667
PAT051^c,d^	41	14	Blast^a^	Mild	45	698	512
PAT062	20	14	Blast^a^	Mild	41	681	618
PAT078^b,c^	46	14	MVA	Severe	46	753	608
PAT081^d^	25	14	Fall	Mild	–	1109	555
PAT101	28	13	Blast	Mild	47	828	500
PAT106^d^	25	14	Blast	Mild	57	870	537
PAT109	29	10	Blast	Mild	54	958	482
PAT110^c,d^	47	14	Blast^a^	Mild	52	914	601
PAT111	28	12	Fall	Mild	43	892	451
PAT112^c^	29	14	Blast	Mild	27	831	493
PAT113^d^	61	16	MVA^a^	Mild	52	831	493
PAT114^c,d^	27	14	Blast	Mild	72	621	490
PAT115^c,d^	48	13	Blast	Mild	59	633	536
PAT117^c^	49	12	Fall	Mild	47	957	608
PAT120^c^	28	14	Fall	Mild	68	603	521
PAT122^c,d^	39	16	MVA	Mild	64	864	529
PAT123^c,d^	25	12	Blast^a^	Mild	72	1057	486
PAT125^c,d^	23	14	Fall	Mild	67	957	491
PAT143^c,d^	29	14	Fall	Mild	47	734	538

*TBI, traumatic brain injury; PCL, PTSD Checklist score. Age in years. Edu, years of education; MVA, moving vehicle accident. ^a^Multiple TBIs; ^b^female; ^c^chronic pain; ^d^sleep problems.*

#### Materials and Procedures

The methods were identical to those of the first session of Experiment 1.

#### Timing Precision

Event-time uncertainties for 6,225 stimulus presentations averaged 0.12 ms (0.04 ms), with a maximal uncertainty of 0.9 ms. Uncertainties for 3,559 responses averaged 0.17 ms (±0.09 ms), with a maximal uncertainty of 0.9 ms.

#### Statistical Analysis

Results from Experiment 3 were compared to the normative population data using the age and computer-use regression functions established in Woods et al. (2015c). In addition, we compared the performance of the TBI patients with the performance of participants in the first test session of Experiment 1, and the simulated malingerers in Experiment 2.

### Results

A summary of the results of Experiment 3 is included in **Table 2**. In comparison with the normative controls, omnibus *z*-scores were increased in the TBI patient group [0.56, *F*(1,1490) = 7.97, *p* < 0.005, Cohen’s *d* = 0.48], but increases in log-mSOA *z*-scores showed only a trend [0.33, *F*(1,1490) = 3.98, *p* < 0.05, Cohen’s *d* = 0.33]. A comparison of the TBI group with the participants in Experiment 1A showed increases in both omnibus *z*-scores [*F*(1,70) = 10.69, *p* < 0.005, $\omega_{p}^{2}$ = 0.12] and log-mSOA *z*-scores [*F*(1,70) = 9.38, *p* < 0.005, $\omega_{p}^{2}$ = 0.11]. However, the increase in CRT *z*-scores in the TBI patients (mean *z*-score = 0.42, ±1.25) did not reach significance either in comparison with the normative group [*F*(1,1490) = 3.53, *p* < 0.06, Cohen’s *d* = 0.37] or in comparison with Experiment 1A participants [*F*(1,70) = 1.55, NS]. CentPT *z*-scores were also similar in the TBI patients and controls [*F*(1,1490) = 2.01, *p* < 0.16 vs. normative controls; *F*(1,70) = 2.87, *p* < 0.10 vs. the controls in Experiment 1A]. As in controls, CentPTs in the TBI patients correlated very strongly with CRTs (*r* = 0.96).

Stimulus-type and compatibility effects did not differ significantly from those seen in control participants; ANOVAs with Group (TBI patients vs. normative controls), Stimulus-type, and Stimulus-Response Spatial-Compatibility as factors showed no significant Group differences in Stimulus-Type or Spatial-Compatibility for either CRT or hit rate measures in comparisons with either the normative control group or the participants in Experiment 1A.

#### Simulated Malingerers vs. TBI Patients

Simulated malingerers performed more poorly than TBI patients, showing greater elevations in omnibus *z*-scores [*F*(1,70) = 43.74, *p* < 0.0001, $\omega_{p}^{2}$ = 0.38] and log-mSOA *z*-scores [*F*(1,70) = 32.84, *p* < 0.0001, $\omega_{p}^{2}$ = 0.31], and increased CRT *z*-scores [*F*(1,70) = 9.64, *p* < 0.003, $\omega_{p}^{2}$ = 0.11]. In contrast, CentPT *z*-scores were significantly reduced in the simulated malingerers when compared to the TBI patients [*F*(1,70) = 24.31, *p* < 0.0001, $\omega_{p}^{2}$ = 0.25], and latency-consistency *z*-scores were correspondingly elevated [*F*(1,70) = 42.24, *p* < 0.0001, $\omega_{p}^{2}$ = 0.54].

#### The Effects of sTBI and mTBI

Omnibus *z*-scores were increased in sTBI patients in comparison with the normative control group [*F*(1,1468) = 7.04, *p* < 0.0001, Cohen’s *d* = 1.05] and Experiment 1A participants [*F*(1,48) = 8.85, *p* < 0.005, $\omega_{p}^{2}$ = 0.14], while mTBI patients showed *z*-score elevations (0.44) that only trended toward significance [versus normative controls *F*(1,1486) = 4.13, *p* < 0.05, Cohen’s *d* = 0.39; versus Experiment 1A controls, *F*(1,66) = 6.52, *p* < 0.02, $\omega_{p}^{2}$ = 0.08]. However, although omnibus *z*-score abnormalities were larger in sTBI than mTBI patients, the inter-group difference failed to reach significance [*F*(1,24) = 1.73, NS].

### Discussion

The TBI patient group showed small but significant impairments in omnibus *z*-scores, largely reflecting increases in log-mSOA *z*-scores. Increases in CRT *z*-scores were also evident, but only trended toward significance. We found no differences in the effects of Stimulus-Type and Stimulus-Response Spatial Compatibility in TBI patients and controls. This suggests that TBI patients experienced the same relative increase in difficulty with harder-to-discriminate distractors and stimulus-response incompatibility as controls. Significant omnibus *z*-score elevations were seen in five TBI patients, all of whom had latency-consistency *z*-scores within the normal range. This pattern was opposite to that seen in the simulated malingerers, who generally showed much larger increases in latency-consistency *z*-scores than omnibus *z*-scores.

#### The Effects of Simulated Malingering and TBI

Consistent with previous results (Willison and Tombaugh, 2006), subjects in simulated malingering conditions performed significantly more poorly than the patients with TBI. The latency-consistency *z*-score > 3.0 cutoff developed in Experiment 2 correctly assigned 96% of TBI patients to the non-malingering group. The latency-consistency metric developed in Experiment 2 also detected two mTBI patients identified in previous studies as performing with suboptimal effort. Both of these patients showed large elevations in omnibus *z*-scores (4.89 and 3.02). Had these patients been included in the mTBI patient group, group-level differences with control populations would have been substantially enlarged. This highlights the importance of incorporating performance-validity metrics in studies of TBI patient populations (Russo, 2012), even when patients are volunteers who are informed in advance that their results will not influence clinical diagnoses or existing pension claims.

#### Limitations

The effect sizes of group comparisons were relatively modest, even for omnibus *z*-scores, indicating that TBI had relatively subtle effects on performance. The TBI patient sample was small, particularly the sTBI patient group, thus limiting the statistical significance of the results as well as the sensitivity to differences between sTBI and mTBI patient groups. Finally, the degree to which mTBI results may generalize to civilian mTBI patients remains to be determined. Abnormalities in the veteran patients tested here may have been increased because most of the patients had a history of blast exposure (Kontos et al., 2013) and concomitant symptoms of PTSD, which can also exacerbate performance deficits (Verfaellie et al., 2014).

## General Discussion

### Cognitive Processes and CRT Test Performance

Performance on the CRT test depends on the speed and accuracy of visual discrimination, response selection, and motor response output. Increasing demands are placed on sustained attention toward the end of the test, as SOAs decrease. While age-related changes are primarily evident in slowed response selection and production (Woods et al., 2015c), deficits in patients with TBI primarily reflect increased errors, which may reflect difficulties in maintaining sustained attention.

### Differences between the CRT Test and Other Choice Reaction Time Tests of Processing Speed

The CRT test is a reliable 6-min test of processing speed that can be downloaded at www.ebire.org/hcnlab/cognitive-tests/CRT. The CRT test produced similar CRTs and age-related changes in performance in two previous large-scale studies (Woods et al., 2015d). In contrast to existing CRT paradigms, the CRT test permits comparisons of CRTs to stimuli presented in the left vs. right hemifield (for use in studies of patients with callosal and unilateral brain lesions), and also permits the analysis of the effects of stimulus discriminability and stimulus-response compatibility on performance.

The CRT test was found to be sensitive in detecting simulated malingerers based on inconsistencies between CentPT and CRT measures. In particular, a latency-consistency *z*-score cutoff of 3.0 discriminated whether abnormal performance was due to suboptimal effort (i.e., in simulated malingerers) or organic causes (i.e., in slow control participants and TBI patients) with 87% sensitivity and 96% specificity. This suggests that the CRT test not only provides useful information about processing speed and attention, but is also a sensitive performance-validity test that can detect suboptimal effort.

## Conclusion

Visual CRTs in a rapid serial visual feature-conjunction test were studied in three experiments. Experiment 1 investigated the effects of repeated testing in a highly educated, young control group whose initial performance was well-predicted by regression functions obtained in a normative population of control participants. ICCs were high for both CRT (0.90) and omnibus *z*-score (0.81) performance measures, which evaluated both processing speed and accuracy. Performance improved significantly over three test sessions. Experiment 2 studied the same participants when instructed to feign symptoms of TBI. More than 87% of the simulated malingerers showed abnormal performance. A latency-consistency *z*-score metric accurately discriminated simulated malingerers from controls with 87% sensitivity and 99% specificity, including 96% specificity when considering only controls with abnormal performance. Experiment 3 found small but significant performance deficits in 26 military veterans with TBI that were more severe in patients who had suffered severe rather than mild TBI. The CRT test reveals the effects of learning, simulated malingering, and TBI on the speed and accuracy of visual stimulus processing.

## Conflict of Interest Statement

David L. Woods is affiliated with NeuroBehavioral Systems, Inc., the developers of Presentation software that was used in these experiments. The other authors declare that the research was conducted in the absence of any commercial or financial relationships that could be construed as a potential conflict of interest.

## Footnotes

1 This test occurred midway through a test session that included the following computerized tests and questionnaires: finger tapping, simple reaction time, Stroop interference, forward and backward digit span, fluency, the Wechsler Test of Adult Reading (WTAR), visual feature conjunction, risk and loss avoidance, delay discounting, the Paced Auditory Serial Addition Task (PASAT), the Cognitive Failures Questionnaire (CFQ), the Posttraumatic Stress Disorder Checklist (PCL) and a traumatic brain injury (TBI) questionnaire.

2 CRT test software and installation instructions can be downloaded at www.ebire.org/hcnlab.
